# Cancer Appetite and Symptom Questionnaire (CASQ) for Brazilian Patients: Cross-Cultural Adaptation and Validation Study

**DOI:** 10.1371/journal.pone.0156288

**Published:** 2016-06-08

**Authors:** Maria Claudia Bernardes Spexoto, Sergio Vicente Serrano, Vanessa Halliday, João Maroco, Juliana Alvares Duarte Bonini Campos

**Affiliations:** 1 Departamento de Alimentos e Nutrição, Faculdade de Ciências Farmacêuticas de Araraquara – UNESP – Univ Estadual Paulista, Rod. Araraqura-Jaú, km 01, Araraquara, São Paulo, Brazil; 2 Barretos Cancer Hospital - Fundação Pio XII - Barretos, São Paulo, Brazil; 3 Public Health, School of Health and Related Research, University of Sheffield, Sheffield, United Kingdom; 4 Departamento de Ciências Psicológicas e Unidade de Investigação em Psicologia e Saúde, ISPA-IU, Rua Jardim do Tabaco, n°34, 1149–041, Lisboa, Portugal; 5 Departamento de Alimentos e Nutrição, Faculdade de Ciências Farmacêuticas de Araraquara – UNESP – Univ Estadual Paulista, Rod. Araraquara-Jaú, km 01, Araraquara, São Paulo, Brazil; The James Cook University Hospital, UNITED KINGDOM

## Abstract

**Background:**

Appetite and symptoms, conditions generally reported by the patients with cancer, are somewhat challenging for professionals to measure directly in clinical routine (latent conditions). Therefore, specific instruments are required for this purpose. This study aimed to perform a cultural adaptation of the Cancer Appetite and Symptom Questionnaire (CASQ), into Portuguese and evaluate its psychometric properties on a sample of Brazilian cancer patients.

**Methods:**

This is a validation study with Brazilian cancer patients. The face, content, and construct (factorial and convergent) validities of the Cancer Appetite and Symptom Questionnaire, the study tool, were estimated. Further, a confirmatory factor analysis (CFA) was conducted. The ratio of chi-square and degrees of freedom (χ^2^/df), comparative fit index (CFI), goodness of fit index (GFI) and root mean square error of approximation (RMSEA) were used for fit model assessment. In addition, the reliability of the instrument was estimated using the composite reliability (CR) and Cronbach’s alpha coefficient (α), and the invariance of the model in independent samples was estimated by a multigroup analysis (Δχ^2^).

**Results:**

Participants included 1,140 cancer patients with a mean age of 53.95 (SD = 13.25) years; 61.3% were women. After the CFA of the original CASQ structure, 2 items with inadequate factor weights were removed. Four correlations between errors were included to provide adequate fit to the sample (χ^2^/df = 8.532, CFI = .94, GFI = .95, and RMSEA = .08). The model exhibited a low convergent validity (AVE = .32). The reliability was adequate (CR = .82 α = .82). The refined model showed strong invariance in two independent samples (Δχ^2^: λ: p = .855; i: p = .824; Res: p = .390). A weak stability was obtained between patients undergoing chemotherapy and radiotherapy (Δχ^2^: λ: p = .155; i: p < .001; Res: p < .001), and between patients undergoing chemotherapy combined with radiotherapy and palliative care (Δχ^2^: λ: p = .058; i: p < .001; Res: p < .001).

**Conclusion:**

The Portuguese version of the CASQ had good face and construct validity and reliability. However, the CASQ still presented invariance in independent samples of Brazilian patients with cancer. However, the tool has low convergent validity and weak invariance in samples with different treatments.

## Introduction

Cancer patients may report changes in appetite [1]. These changes may be described as a lack of desire to eat, a change in the taste of food, and a perception of early satiety [1–3]. Changes in appetite may manifest as changes in weight [4, 5]. Contributing factors include digestive dysfunctions such as nausea, vomiting, and constipation. Because of the complexity of underlying factors contributing to changes in appetite, it can be important to assess appetite and related symptoms of digestive disturbance to best guide clinical care.

Both the loss of appetite and the weight loss are common and troubling characteristics related to cancer patients, especially to patients with cancer in advanced stages, patients refractory to treatment, or patients without treatment options [2]. Quinten et al. [6] evaluated appetite and its relationship to survival in 1,314 patients with cancer, and found that patients with better appetite lived longer.

After evaluating 3,047 cancer patients, Dewys et al. [7] found that symptoms such as nausea, loss of appetite, and diarrhea can contribute significantly to weight loss. Additionally, the authors found a significant reduction in the average survival time of patients with low weight, as compared to those with normal weight, regardless of the tumor’s location. Subsequent studies have confirmed these findings [8–10].

It should be emphasized that appetite can be affected either by the disease, or by the treatment, which can cause symptoms such as nausea, vomiting, constipation, and changes in taste or pain [11–13].

Given the importance that appetite has on the response to several treatments [7] and on the disease progression [8, 10], several psychometric instruments to measure it have been proposed [14–17]. Among these, the Functional Assessment of Anorexia/Cachexia Therapy (FAACT) questionnaire [14], the Appetite, Hunger and Sensory Perception (AHSP) questionnaire [15], the Council on Nutrition Appetite Questionnaire (CNAQ) [16] and the Simplified Nutrition Appetite Questionnaire (SNAQ) [16, 17] stand out.

Considering that appetite is a latent condition, i.e., not directly measurable, it is necessary to evaluate the psychometric properties of data gathered with this instrument before using it, by assessing its validity and reliability. Only then the data can be used with confidence ensuring the validity of the results and conclusions reached with that data [18, 19].

To assess appetite and symptoms specifically in cancer patients, Halliday et al. [20] proposed the *Cancer Appetite and Symptom Questionnaire* (CASQ). The CASQ is a one-factor instrument comprising 12 items that allow responses on a five-point Likert scale. The CASQ was proposed in English, for the UK population, and so far does not have versions in any other language. The CASQ was adapted from the *Council on Nutrition Appetite Questionnaire* (CNAQ) [16] and four items were added to meet the characteristics of cancer patients [20].

Thus, this study aimed to develop a cultural adaptation of the CASQ, into Portuguese, and to evaluate its psychometric properties on a sample of Brazilian cancer patients in curative and palliative treatments.

## Method

### Study Design

The cross-cultural adaptation and validation of the Appetite and Symptom Questionnaire involved three phases: Phase 1 (Transcultural adaptation), Phase 2 (Content validity), and Phase 3 (Evaluation of the psychometric characteristics).

### Phase 1: Transcultural Adaptation of the CASQ

#### Face Validity

To confirm face validity of the CASQ we used the methods proposed by Guillemin et al. [21] and Beaton et al. [22]. The instrument was translated independently by three bilingual translators who were native speakers of Portuguese, had English knowledge, and had lived in an English speaking country. The translations were evaluated by the researchers of the present study, in order to obtain consensus on a single Portuguese version. This version was back-translated by a bilingual translator whose mother tongue was English and compared with the original CASQ.

The Portuguese version was then pre-tested on a group of 32 cancer patients to verify the Misunderstanding Index (MI) of each item of the CASQ. Given that no item presented an MI > 20%, it was not necessary to change any words and/or the grammatical construction of the items. The Portuguese version was then evaluated by a team comprising three Portuguese teachers and three experts in oncology to verify the semantic, idiomatic, cultural, and conceptual equivalence of the instrument to the original version.

### Phase 2: Content Validity

The content validity of the CASQ was estimated utilizing the method proposed by Lawshe [23]. During this stage, 12 judges who were experts in the field of Oncology and Nutrition rated each item on the instrument according to its essentiality (“essential,” “useful, but not essential,” and “not necessary”). The Content Validity Ratio (CVR) was computed, and the significance was assessed according to the method proposed by Wilson et al. [16], adopting a significance level of 5% (CVR_12; .05_ ≥ 0.57). This stage complements and/or helps to decide whether to remove/maintain instrument items. This decision can only be made after confirmatory factor analysis, that is, when items with low factor weight are present or where there is difficulty of adjusting the model to the sample (which is verified after assessment of all psychometric properties), the items associated with this difficulty are identified and CVR is used for decision making.

### Phase 3: Evaluation of Psychometric Characteristics

Phase 3 was a cross-sectional study, with a non-probabilistic convenience sampling design.

#### Participants

Patients attending the Barretos Cancer Hospital outpatient and inpatient clinics with a diagnosis of malignant neoplasms were invited to take part. The sample selection was performed for convenience (non-probabilistic). Those undergoing major and intermediate complex surgical procedures within 30 days of the interview, with cognitive impairment or severe psychiatric disorders, and under the age of 18 years were excluded from the sample. The study included only cancer patients who agreed and signed the free and informed consent form.

#### Sample size calculation

The estimated minimum sample size was based on the requirement of 10 subjects per model parameter [24]. Given that the instrument (CASQ) had 24 parameters, the required sample size was 240. As the invariance of the instrument in two independent samples was also evaluated in this study, it was necessary to use a second sample with the same size. Therefore, the estimated minimum necessary sample size was 480 participants. Given that this study also aimed to examine the psychometric qualities of the CASQ on cancer patients in Brazil, it was considered that the sample was large enough to conveniently capture the variability in this population [18]. Thus, we chose to work with a representative sample bigger than that recommended for the statistical analysis.

#### Study variables and instrument

To characterize the sample, demographics and clinical information were collected. The socio-demographic data collected included gender, age, marital status, presence of any religious practices, work-related activity, number of people in the household, and socioeconomic status and education of the head of the household. Age was assessed in years; marital status in categories such as single, married, widowed, and separated/divorced. The religion and its practice, and work-related activity were assessed dichotomously (presence/absence). The economic and educational level of the head of the household were classified according to the Brazil Criterion—ABEP [25].

Clinical information regarding the disease was obtained by consulting the patients’ clinical record. The variables evaluated were the presence of a definite diagnosis (presence/absence), type of neoplasm, stage, treatment type (chemotherapy, radiotherapy, chemotherapy and radiotherapy, hormone therapy, and immunotherapy and palliative care) and metastasis (presence/absence). Information on the location of the patient at the time of assessment (outpatient or inpatient) was also collected.

The height (cm) and weight (kg) reported by the patient were recorded for calculating the body mass index (kg/m^2^) (BMI). To determine the classification of BMI, the cutoff points for adults (> 20 years), proposed by the World Health Organization [26], were used.

Symptoms and appetite was assessed using the Cancer Appetite and Symptom Questionnaire (CASQ) developed by Halliday et al [20]. It should be clarified that four items of the instrument had a reversed response scale. The author’s authorization was acquired before using the instrument.

### Data Analysis

#### Psychometric sensitivity

The summary and shape measures of the CASQ items’ distribution were used to estimate their psychometric sensitivity. Items with a skewness (Sk) greater than 3 and kurtosis (Ku) greater than 7, in absolute values, were considered to have psychometric sensitivity issues [27]. The diagnosis of multivariate outliers was performed by computing the Mahalanobis distance [27].

#### Construct validity

To assess the construct validity of the instruments the factorial and convergent validity were evaluated.

#### Factorial validity

To examine the effectiveness of the adaptation of the CASQ to the study sample, a Confirmatory Factor Analysis was performed using the Maximum Likelihood estimation method implemented in SPSS AMOS (v.22, SPSS an IBM Company, Chigago, IL). To assess the goodness of the model fit to the data variance/covariance matrix, the ratio of chi-square and degrees of freedom (χ^2^/df), comparative fit index (CFI), goodness of fit index (GFI) and root mean square error of approximation (RMSEA) were used [19]. The fit of the model was considered adequate when χ^2^/df ≤ 2.0, CFI and GFI ≥ .90 and RMSEA ≤ .08 for each sample [19, 24].

Items that had factor weights (λ) < .30 were removed, as well as those that proved redundant by the modification indices, estimated through the Lagrange multipliers (LM > 11, p < .001). The modification indices were also used to verify the correlation between the items’ errors [19].

To define the best model (complete or refined) the indices based on information theory were used, namely, the Akaike Information Criterion (AIC), Bayes Information Criterion (BIC), and Browne-Cudeck Criterion (BCC), and the model that presented lower values in one or more of these indices was considered the most parsimonious.

#### Factorial Invariance

To verify the invariance of the factor structure obtained, a cross-validation of the model was performed by means of a multigroup analysis. For this, the sample was randomly divided into two parts (6:4), 60% comprised the “Test Sample” and 40% the “Validation Sample.” The invariance of the models was tested using the chi-square difference (Δχ^2^) statistics between the two models. The model was considered invariant when Δχ^2^ p>0.05. The invariance of: i) factor weights (λ) (metric invariance/weak invariance), ii) factor weights (λ) and items intercepts (i) (scalar invariance/strong invariance), iii) factor weights (λ), items intercepts (i) and residuals variances/covariances (residuals invariance/strict invariance) (Res) were tested [28].

The invariance of the factor model was also tested by subdividing the participants according to treatment type (chemotherapy, radiotherapy, chemotherapy and radiotherapy, and palliative care).

#### Convergent validity

The Average Variance Extracted (AVE) was used to assess the convergent validity of the CASQ [19, 24]. Acceptable values (AVE ≥ .50) are indicative of the factor’s convergent validity) [24].

#### Reliability

The reliability of the CASQ was estimated by the Composite Reliability (CR) and the standardized Cronbach’s coefficient alpha (α), which were considered adequate when CR α ≥ .70 [29, 30].

#### Calculation of the appetite and symptom global score

After fitting the model to the sample variance/covariance matrix data, the overall score for the “Appetite and Symptoms” was calculated using a matrix of factor score weights produced by the fitted model [19]. This weight was assigned to each item and was multiplied by the answer given by each participant, after which all weighted items were summed, obtaining an overall score.

All analyses were performed using the IBM SPSS Statistics (v.22, SPSS An IMB Company, Chicago, IL) and AMOS 22.0 (SPSS An IMB Company, Chicago, IL) programs.

### Ethical Considerations

This study followed the ethical principles of Resolution 466/12 of the National Board of Health, and was approved by the Research in Human Beings’ Ethics Committee of the Barretos Cancer Hospital (Barretos-São Paulo) (protocol 561/2011).

## Results

### Face Validity

At the stage of face validity, the pre-test evaluation, there was a need to change only item 12 (presence and / or severity of pain), more specifically in its response scale. This change was explained by the need to identify of Individuals without pain. Therefore, the response scale to that item was modified to 6 points: “no pain,” “very light,” “mild,” “moderate,” “severe,” and “very severe” (thus, including the response no pain). No other adaptation was necessary.

### Participants

A total of 1,219 patients with cancer, treated at the Cancer Hospital of Barretos (São Paulo - Brazil) in 2013, were invited to participate. Of these 1,140 (93.5%) agreed to participate.

The reasons for not participating in to the survey were lack of time (n = 5), having already participated in another study on the same day (n = 1), shyness (n = 2), not feeling well (n = 3), refusal to fill out the demographic questionnaire (n = 1), no justification (n = 7), not wanting to participate given that data collection was in an interview format (n = 60).

The mean age of the participants was 53.95 (SD = 13.25) years and only 1 patient was reported to have no definite diagnosis. The sociodemographic and clinical characterization of the participants has been presented in Table 1. It is important to note that not all patients answered all the questions of the sociodemographic inventory (“religion” n = 16; “religion practice” n = 15; “marital status” n = 2; “work activity” n = 3) and some clinical information was not included in clinical records (“data collection location” n = 7; “stage" n = 33; “type of treatment” n = 4; “presence of metastasis” (n = 6) and “Body Mass Index” n = 16).

**Table 1:** Sociodemographic and Clinical Characterization of the study Participants.

Sociodemographic characteristics	n	%	Clinical Characteristics	n	%
**Gender**			Specialty of the Diagnosis*		
Male	441	38.7	Head and Neck	79	6.9
Female	699	61.3	Upper digestive tract	108	9.5
**Religion**			Lower digestive tract	222	19.5
No	49	4.4	Gynecology	135	11.8
Yes	1075	95.6	Hematology	5	0.4
**Religious Practice**			Breast Cancer	335	29.4
No	156	13.9	Brain Tumor	17	1.5
Yes	969	86.1	Orthopedics	27	2.4
**Marital status**			Skin	46	4
Single	155	13.6	Thorax^#^	66	5.8
Married	740	65.0	Urology	100	8.8
Widowed	128	11.2	**Stage**		
Separated/Divorced	115	10.1	I	81	8.0
**Work Activity**			II	233	23.1
No	843	74.1	III	371	36.8
Yes	294	25.9	IV	322	32.0
**Economic Class**			**Type of treatment**		
A	27	2.4	Chemotherapy	649	57.1
B	393	34.5	Radiotherapy	171	15.1
C	536	47.0	Chemotherapy and radiotherapy	150	13.2
D and E	184	16.2	Hormone-therapy	26	2.3
**Data collection location**			Immunotherapies	20	1.8
Outpatient consultation	1051	92.8	Palliative care	120	10.6
Hospitalization Units	82	7.2	**Presence of metastasis**		
			No	627	55.3
			Yes	507	44.7
			**Body Mass Index (BMI)**		
			< 18.5 (Low weight)	78	6.9
			18.5–25.0(Normal)	468	41.6
			25.0–30.0 (Pre-obesity)	363	32.3
			≥ 30.0 (Obesity)	215	19.1

* The criterion adopted was based on the classification presented by the Barretos Cancer Hospital following the subspecialties of Clinical Oncology

^#^ Lung, pleura and mediastinum

### Psychometric sensitivity and Content Validity

Table 2 presents the summary measures of the items of the Appetite and Symptoms Questionnaire for Cancer Patients Portuguese version, for patients with cancer, and the Content Validity Ratio (CVR).

**Table 2:** Summary Measures and Content Validity Ratio (CVR) for the Items of the Portuguese Version of the Appetite and Symptoms Questionnaire for Cancer Patients (CASQ).

CASQ	Median	Mean	Mode	Standard Deviation	Skewness	Kurtosis	CVR*
It 1	1	1.55	1	1.15	0.44	-0.55	1.00
It 2	0	0.68	0	1.18	1.60	1.19	.33^a^
It 3	3	2.34	3	1.33	-0.38	-1.04	.00^a^
It 4	0	0.62	0	1.11	1.64	1.50	.33^a^
It 5	1	1.05	1	0.80	0.69	0.82	.83
It 6	3	2.71	3	1.02	-0.73	0.10	-.50^a^
It 7	2	2.45	2	0.73	0.27	0.66	.83
It 8	0	0.90	0	1.19	1.10	0.01	.67
It 9	0	0.73	0	1.14	1.48	1.24	.67
It 10	1	1.50	1	0.86	0.40	0.10	.00^a^
It 11	2	1.87	2	1.00	0.08	-0.27	.67
It 12	0	0.84	0	1.16	1.15	0.22	1.00

* CVR_12; .05_ ≥ .57

^a^values below the minimum significant CVR

No items presented severe values of Sk and Ku, which indicates the adequate psychometric sensitivity of the items. In the opinion of the judges/experts, 5 items (it2. When I eat I feel full, it3. Before eating, I feel hungry, it4. I enjoy the food I do eat, it6. At present I eat in addition to or instead of meals, it10. Most of the time my mood is) of the CASQ were not essential for the assessment of appetite and symptoms.

### Factorial and Convergent validity and Reliability

**Fig 1:**
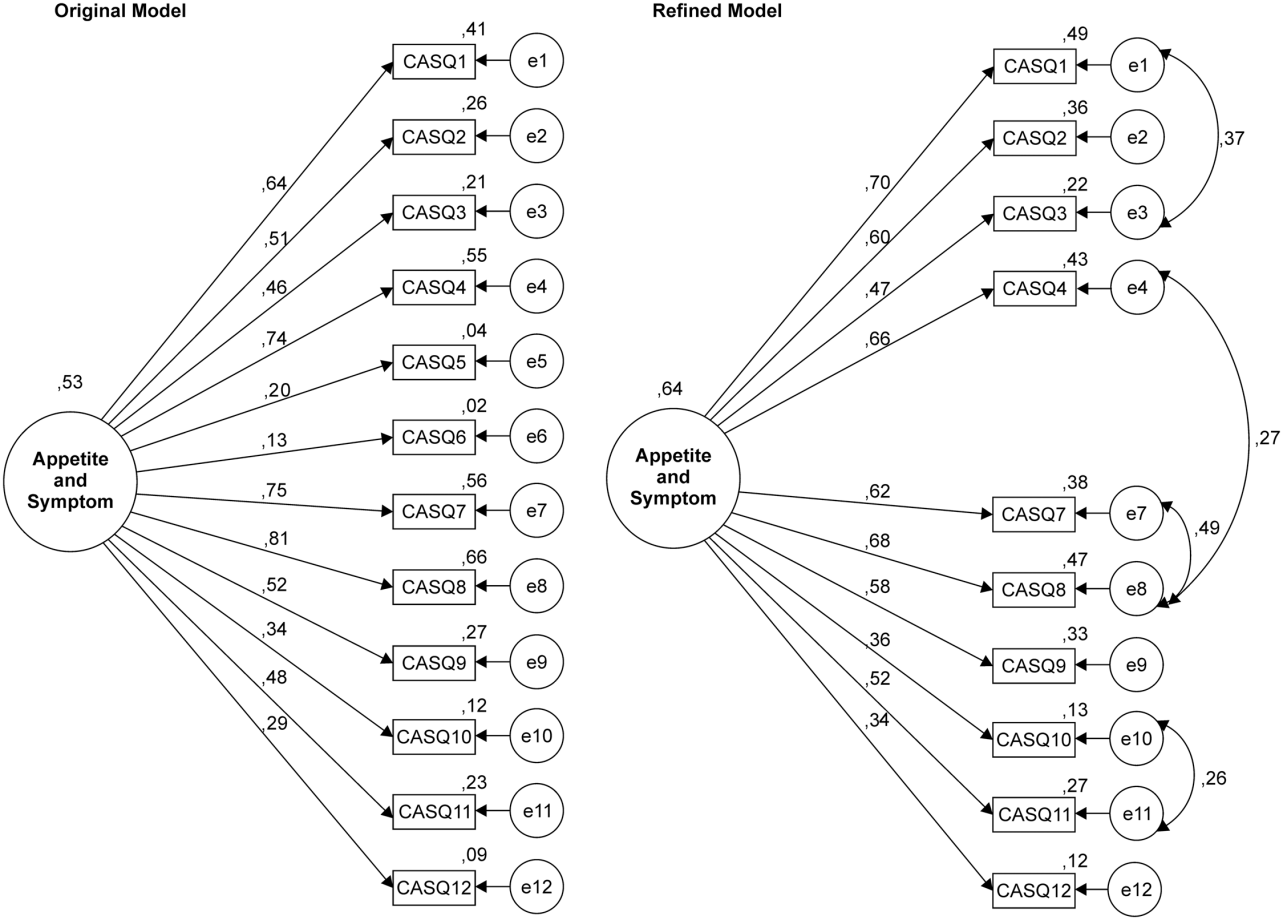
Factor structure of the original model CASQ. Factor structure of the original model (CFA: λ = .13–.81; χ^2^/df = 18.002, CFI = .761, GFI = .846 RMSEA = .122, AIC = 1,020.112, BCC = 1,020.666, BIC = 1,141.043, σ^2^ = 0,53) and the refined model fitted to the Brazilian sample of patients with cancer (CFA: λ = .34–.70; χ^2^/df = 8.532, CFI = .936, GFI = .954, RMSEA = .081, AIC = 312.505, BCC = 312.973, BIC = 433.436, σ^2^ = 0,64).

The confirmatory factor analysis (CFA) of the original CASQ structure and refined model fitted to the Brazilian sample of patients with cancer is presented in Fig 1.

Two items presented inadequate factor weights (λ < .30). The fit of the original model to the sample was unsatisfactory. For the CASQ to present an adequate fit to the sample, items with λ < .30 were removed, and four correlations between errors were included (e1–e3, e4–e8, e7–e8, e10–e11). The refined model presented adequate fit to sample and explained 64% of the variance. Thus, the average variance extracted was below of the adequate (AVE = .32). The reliability was adequate (CR and α = .82).

Table 3 presents the findings of the confirmatory factor analysis (CFA), average variance extracted (AVE), composite reliability (CR) and internal consistency (α) of the refined CASQ model fitted to different samples.

**Table 3:** The Confirmatory Factor Analysis (CFA), Average Variance Extracted (AVE), Composite Reliability (CR) and Internal Consistency (α) of the Refined Model CASQ Fitted to Different Samples.

			CFA*							
Sample	n	λ	χ^2^/df	CFI	GFI	r_errors_	RMSEA	AVE	CR	α
Test	695	.33–.73	5.99	.93	.95	.25–.47	.09	.33	.83	.81
Validation	445	.34–.66	4.21	.93	.94	.27–.52	.08	.30	.81	.79
Chemotherapy	649	.31–.69	4.79	.94	.95	.26–.47	.08	.29	.79	.78
Radiotherapy	171	.37–.76	3.23	.90	.89	.34–.54	.11	.35	.83	.83
Chemotherapy and Radiotherapy	150	.31–.82	1.59	.97	.94	.10–.56	.06	.38	.85	.84
Palliative	120	.10–.80	1.58	.94	.92	.22–.34	.07	.30	.79	.78

*CFA: λ = factor weight, χ^2^/df = Ratio chi-square by the degrees of freedom, CFI = *Comparative of Fit Index*, GFI = G*oodness of Fit Index*, r_errors_ = correlation between errors, RMSEA = *Root Mean Square Error of Approximation*; AVE = Average Variance Extracted; CR = Composite Reliability, α = Standardized Cronbach’s alpha.

The refined model presented an adequate fit in all the tested samples, except for the sample submitted to radiotherapy (RMSEA = .11). However, it is important to emphasize that a low factor weight (λ = .10) was exhibited by the sample of palliative care patients for the item regarding the presence/severity of pain (it 12).

The model presented low convergent validity, and the reliability (CR and α) was adequate.

### Appetite and symptom global score

After verifying the adequacy of the refined model to the sample, we determined the algorithm for calculating the overall Appetite and Symptom score in the sample, as presented in Eq 1.

$$\begin{array}{l} Apettite=.171it1+.125it2+.026it3+.138it4+.153it7+\\ +.084it8+.117it9+.047it10+.101it11+.053it12 \end{array}$$ (1)

For each item of the CASQ the scale of responses is scored from 0 to 4 and, in order to obtain the overall CASQ score for each participant, the response given to each item should be summed. In addition, strategies for evaluating the score obtained may be implemented. For example, use of percentiles 25, 50 and 75 of the scale where a score ≤ 1 represents low impairment of the Appetite/ Symptoms, 1 to 3 moderate impairment and a score > 3 severe impairment. We believe that the Appetite/Symptoms classification could strategically be adjusted by clinicians to reflect the level of their involvement in clinical practice.

### Factorial Invariance

After verifying the best fit of the CASQ refined model to the sample, the invariance of the model was evaluated in independent samples (Table 4).

**Table 4:** Multigroup Analysis of the CASQ’s refined model on independent samples.

	Δχ^2^^#^		
Groups	λ	I	Res
Test × Validation	4.75 (.855)	5.89 (.824)	15.88 (.390)
Chemotherapy × Radiotherapy	13.17 (.155)	88.66 (<.001)	53.63 (<.001)
Chemotherapy × Chemotherapy and Radiotherapy	8.12 (.522)	24.12 (.007)	58.54 (<.001)
Chemotherapy × Palliative Care	27.56 (.001)	145.22 (<.001)	243.37 (<.001)
Radiotherapy × Chemotherapy and Radiotherapy	21.44 (.011)	53.46 (<.001)	98.69 (<.001)
Radiotherapy × Palliative Care	40.16 (<.001)	158.71 (<.001)	194.69 (<.001)
Chemotherapy and Radiotherapy × Palliative Care	16.48 (.058)	90.87 (<.001)	63.78 (<.001)

^#^ Δχ^2^:λ = factor weight, i = factor weight and items intercept; Res = residuals

The refined model showed strong invariance in the independent samples (Validation x Test) and weak invariance in three subsamples (Chemotherapy × Radiotherapy, Chemotherapy × Chemotherapy and Radiotherapy, Chemotherapy and Radiotherapy × Palliative Care). The invariance of the factor structure was not similar among the treatment types.

The Portuguese version of CASQ is given in S1 Appendix.

## Discussion

The refined model showed strong invariance in independent samples (Validation × Test). The invariance of the factor structure was not similar among the different treatment types.

For the structure of the CASQ to adequately fit the sample variance/covariance matrix, it was necessary to remove Items 5 and 6 (Fig 1). One may speculate that, the low factor weights found may be related with the difficulty in interpreting the term “meal.”

The term “meal” in Brazil often refers to a ritual of socialization that includes elements such as the use of knife and fork, and sitting at the table [31]. In this sense, responses to this item may have been influenced by this connotation, i.e., individuals may have had difficulty in understanding that the concept of meal included any process of feeding and not only those situations involving the elements outlined above. However, it must be clear that this justification was based on the reflection of the researchers on the low factor weights found and the theoretical construct regarding items 5 and 6. The respondents did not mention this difficulty in the pilot study, which prevented changing the term “meal” during the creation of the Portuguese version of the CASQ. This manifestation appeared in the final study where most respondents (99.4%) understood the term meal as a ritual (table, fork, knife…), not considering the entire process of consuming foods throughout the day (for example: snacks between meals). This fact can also be confirmed by the answer pattern of items 5 and 6. Item 5 presents response bias to lower frequency while item 6 presents the inverse pattern. This difficulty was not observed in the English sample [20], in which the instrument was originally proposed, which is probably due to the cultural differences of the respective samples.

Another aspect to be highlighted is the low factor weight of Item 12, referring to the painful condition, especially in the sample of patients in palliative care (Table 3). This fact can be attributed to the use of medication for effective control of cancer pain in these patients, which may mask the presence and/or severity of the pain [32]. Thus, we suggest caution in the use/interpretation of this item in samples of palliative care patients.

The proposed refined model presented adequate fit in the independent samples according to treatment type (Table 3) and was invariant in the two independent samples and between patients who underwent Chemotherapy and Radiotherapy, Chemotherapy × Chemotherapy and Radiotherapy, Chemotherapy and Radiotherapy × Palliative Care (Table 4). The absence of invariance observed between the models fitted to palliative care patients (Table 4) may be related to clinical differences, both regarding diagnosis and treatment. Patients in palliative care take greater amounts of pain medication, having a greater organic weakness [32] and the feeding process is, in most cases, compromised [33]. This, in turn, makes the assessment of appetite different from that carried out in cancer patients undergoing other treatment modalities. It should be noted however that the lack of invariance in some samples does not prevent the use of the proposed evaluation of Appetite/Symptoms presented in this study for the screening of this condition in patients with cancer. It should be noted that where there is a need to compare specific groups (e.g. other clinical features and/or demographics) a new model of fit should be calculated in order to check the feasibility of using the weights shown in Eq 1, this being due to possible differences in operationalization of the construct. What we have presented is a step by step validation process and algorithm which may be of use to other oncology healthcare professionals.

Another important aspect is the low convergent validity (AVE) (Table 3) observed for the CASQ in all samples, which can be attributed to the high variability found in the factor weights of the items. This variability may suggest the existence of more than one factor for the CASQ, perhaps delimiting Appetite and Symptoms. It should be noted that this study sought to test the original proposal (unifactorial) and that future studies could be developed to evaluate new theoretical proposals, such as, for example, a two-factor structure and/or a second-order hierarchical model. However, it should be remembered that these proposals must be supported by convincing theoretical foundations. We emphasize that the convergent validity limitation did not affect the fit model. Therefore, it did not prevent the use of a single-factor proposal for the assessment of Appetite and Symptoms in cancer patients.

Regarding the evaluation of Appetite and Symptoms, the original version of the CASQ proposes a final score based on the sum of the responses to the items [20]. However, a score derived from the sum of responses does not seem to be the best strategy, since the metric properties of an instrument are influenced by sample characteristics and, therefore, can change in different samples [34, 35].

Thus, we suggest that the overall score is computed using the matrix of factor score weights (Eq 1) [19, 35], which should be adjusted for each sample. Thus, the inclusion or exclusion of items will not affect the calculation of the final score, and it will be more accurate, since the weights of the items are computed for each sample. This preserves the important differences between the items, which can be different for each population. This strategy will result in a more accurate estimate of the score of appetite and symptoms.

Thus, this paper presents the results of a tool for assessment of appetite and symptoms in cancer patients, with adequate psychometric properties, and with an individualized proposal for calculating the final score. This may contribute to a more accurate diagnosis and, thus, a more resolute clinical management strategy.

## Conclusion

The Portuguese version of the CASQ presented good face and construct validity, and reliability. However, the CASQ still presented invariance in two independent samples of Brazilian patients with cancer. It has low convergent validity and weak invariance in samples with different treatments.

## Supporting Information

S1 Appendix The original and Portuguese version of the Appetite and Symptoms Questionnaire for Patients with Cancer (CASQ). (DOCX)

S1 Dataset (SAV)
