# Supplementary material for: Cancer Appetite and Symptom Questionnaire (CASQ) for Brazilian Patients: Cross-Cultural Adaptation and Validation Study
Source: PLoS One. 2016 Jun 8;11(6):e0156288. doi: 10.1371/journal.pone.0156288 (PMC4898714; doi:10.1371/journal.pone.0156288)
Supplement: S1 Appendix — (DOCX) [file pone.0156288.s001.docx]

**Appendix 1.** **The original and Portuguese version of the Appetite and Symptoms Questionnaire for Patients with Cancer (CASQ).**

|  | **Original Version [26]** | | **Portuguese version** | |
| --- | --- | --- | --- | --- |
| **It1** | **My appetite is…** | | **Meu apetite é…** | |
|  | ( ) | very poor | ( ) | Muito pouco |
|  | ( ) | poor | ( ) | Pouco |
|  | ( ) | average | ( ) | Médio |
|  | ( ) | good | ( ) | Bom |
|  | ( ) | very good | ( ) | Muito bom |
| **It2** | **When I eat I feel full…** | | **Quando eu como eu me sinto cheio…** | |
|  | ( ) | without having eaten anything | ( ) | Sem ter comido nada |
|  | ( ) | after eating only a few mouthfuls | ( ) | Depois de comer apenas um pouco |
|  | ( ) | after eating about a third of a meal | ( ) | Depois de comer cerca de um terço duma refeição |
|  | ( ) | after eating over half a meal | ( ) | Depois de comer mais da metade duma refeição |
|  | ( ) | after eating a full meal | ( ) | Depois de comer uma refeição completa |
| **It3** | **Before eating, I feel hungry…** | | **Antes de comer eu sinto fome…** | |
|  | ( ) | rarely | ( ) | Raramente |
|  | ( ) | occasionally | ( ) | Ocasionalmente |
|  | ( ) | some of the time | ( ) | Boa parte do tempo |
|  | ( ) | most of the time | ( ) | A maior parte do tempo |
|  | ( ) | all of the time | ( ) | O tempo todo |
| **It4** | **I enjoy the food I do eat…** | | **Eu gosto da comida que eu como…** | |
|  | ( ) | most times | ( ) | A maioria das vezes |
|  | ( ) | often | ( ) | Frequentemente |
|  | ( ) | sometimes | ( ) | Algumas vezes |
|  | ( ) | rarely | ( ) | Raramente |
|  | ( ) | never | ( ) | Nunca |
| **It5** | **At present I eat…** | | **Atualmente eu como…** | |
|  | ( ) | less than one meal a day | ( ) | Menos de uma refeição por dia |
|  | ( ) | one meal a day | ( ) | Uma refeição por dia |
|  | ( ) | two meals a day | ( ) | Duas refeições por dia |
|  | ( ) | three meals a day | ( ) | Três refeições por dia |
|  | ( ) | more than three meals a day | ( ) | Mais de três refeições por dia |
| **It6** | **At present I eat (in addition to or instead of meals)…** | | **Atualmente eu como (em adição ou em substituição das refeições)…** | |
|  | ( ) | no snacks | ( ) | Nenhum lanche |
|  | ( ) | one snack a day | ( ) | Um lanche por dia |
|  | ( ) | two snacks a day | ( ) | Dois lanches por dia |
|  | ( ) | three snacks a day | ( ) | Três lanches por dia |
|  | ( ) | four or more snacks a day | ( ) | Quatro ou mais lanches por dia |
| **It7** | **Compared to before I was ill, food tastes…** | | **Comparativamente a antes de estar doente, o sabor da comida é…** | |
|  | ( ) | much worse | ( ) | Muito pior |
|  | ( ) | worse | ( ) | Pior |
|  | ( ) | just as good | ( ) | Tão bom quanto antes |
|  | ( ) | better | ( ) | Melhor |
|  | ( ) | much better | ( ) | Muito melhor |
| **It8** | **At present I have…** | | **Atualmente eu tenho…** | |
|  | ( ) | no changes in taste | ( ) | Nenhuma alteração no paladar |
|  | ( ) | mild taste changes | ( ) | Alteração leve no paladar |
|  | ( ) | moderate taste changes | ( ) | Alteração moderada no paladar |
|  | ( ) | severe taste changes | ( ) | Alteração severa no paladar |
|  | ( ) | no taste at all | ( ) | Nenhum paladar |
| **It9** | **I feel sick or nauseated before I eat or when I eat…** | | **Eu me sinto doente ou enjoado antes de comer ou quando como…** | |
|  | ( ) | most times | ( ) | Na maioria das vezes |
|  | ( ) | often | ( ) | Frequentemente |
|  | ( ) | sometimes | ( ) | Algumas vezes |
|  | ( ) | rarely | ( ) | Raramente |
|  | ( ) | never | ( ) | Nunca |
| **It10** | **Most of the time my mood is…** | | **A maior parte do tempo, o meu humor é…** | |
|  | ( ) | very sad | ( ) | Muito triste |
|  | ( ) | sad | ( ) | Triste |
|  | ( ) | neither sad nor happy | ( ) | Nem triste nem feliz |
|  | ( ) | happy | ( ) | Feliz |
|  | ( ) | very happy | ( ) | Muito feliz |
| **It11** | **Most of the time my energy level is…** | | **Na maioria das vezes, o meu nível de energia é…** | |
|  | ( ) | very high | ( ) | Muito alto |
|  | ( ) | high | ( ) | Alto |
|  | ( ) | moderate | ( ) | Moderado |
|  | ( ) | low | ( ) | Baixo |
|  | ( ) | very low | ( ) | Muito baixo |
| **It12** | **Most of the time my pain is…** | | **A maior parte do tempo, minha dor é…** | |
|  |  |  | ( ) | Sem dor |
|  | ( ) | very mild or no pain | ( ) | Muito leve |
|  | ( ) | mild | ( ) | Leve |
|  | ( ) | moderate | ( ) | Moderada |
|  | ( ) | severe | ( ) | Severa |
|  | ( ) | very severe | ( ) | Muito severa |
